# Interactive Temperature and CO_2_ Rise, Salinity, Drought, and Bacterial Inoculation Alter the Content of Fatty Acids, Total Phenols, and Oxalates in the Edible Halophyte *Salicornia ramosissima*

**DOI:** 10.3390/plants12061395

**Published:** 2023-03-21

**Authors:** Jennifer Mesa-Marín, Enrique Mateos-Naranjo, João Carreiras, Eduardo Feijão, Bernardo Duarte, Ana Rita Matos, Marco Betti, Carmen Del Rio, Marina Romero-Bernal, Joan Montaner, Susana Redondo-Gómez

**Affiliations:** 1 Departamento de Biología Vegetal y Ecología, Facultad de Biología, Universidad de Sevilla, 41012 Seville, Spain; 2 MARE—Marine and Environmental Sciences Centre, ARNET—Aquatic Research Infrastructure Network Associated Laboratory, Faculdade de Ciências, Universidade de Lisboa, Campo Grande, 1749-016 Lisbon, Portugal; 3 Departamento de Biologia Vegetal, Faculdade de Ciências, Universidade de Lisboa, Campo Grande, 1749-016 Lisbon, Portugal; 4 BioISI—Biosystems and Integrative Sciences Institute, Plant Functional Genomics Group, Departamento de Biologia Vegetal, Faculdade de Ciências, Universidade de Lisboa, Campo Grande, 1749-016 Lisbon, Portugal; 5 Departamento de Bioquímica Vegetal y Biología Molecular, Facultad de Química, Universidad de Sevilla, 41012 Sevilla, Spain; 6 Institute of Biomedicine of Seville (IBiS), Hospital Universitario Virgen del Rocío, CSIC, Universidad de Sevilla, 41013 Seville, Spain; 7 Department of Neurology, Hospital Universitario Virgen Macarena, 41009 Seville, Spain

**Keywords:** climate change, nutritional quality, plant-growth-promoting rhizobacteria, biofertilizer, gas chromatography, spectrophotometry

## Abstract

In this work, we studied the combined effect of increased temperature and atmospheric CO_2_, salt and drought stress, and inoculation with plant-growth-promoting rhizobacteria (PGPR) on the growth and some nutritional parameters of the edible halophyte *Salicornia ramosissima*. We found that the increase in temperature and atmospheric CO_2_, combined with salt and drought stresses, led to important changes in *S. ramosissima* fatty acids (FA), phenols, and oxalate contents, which are compounds of great importance for human health. Our results suggest that the *S. ramosissima* lipid profile will change in a future climate change scenario, and that levels of oxalate and phenolic compounds may change in response to salt and drought stress. The effect of inoculation with PGPR depended on the strains used. Some strains induced the accumulation of phenols in *S. ramosissima* leaves at higher temperature and CO_2_ while not altering FA profile but also led to an accumulation of oxalate under salt stress. In a climate change scenario, a combination of stressors (temperature, salinity, drought) and environmental conditions (atmospheric CO_2,_ PGPR) will lead to important changes in the nutritional profiles of edible plants. These results may open new perspectives for the nutritional and economical valorization of *S. ramosissima*.

## 1. Introduction

The study of halophytes has increased recently since these crops are an option for sustainable agriculture in marginal environments. The cultivation of conventional crops is facing severe limitations such as scarcity of good quality water and soil salinization and degradation due to climate change and intensive agriculture [1]. Halophytes subsist and reproduce under soil salinities of more than 200 mmol L^−1^ NaCl. In contrast to glycophytes, halophytes are extremely productive under saline irrigation and are able to grow in adverse environments [2]. They are good candidates as cash crops because they may be domesticated through conventional breeding programs, and they still can achieve high, economically lucrative yields [3,4,5,6].

The halophyte *Salicornia ramosissima* J. Woods (Chenopodiaceae) produces succulent shoots and is highly appreciated as a gourmet vegetable [4,6]. *S. ramosissima* shoots are consumed boiled, sautéed, or fresh in salads for their umami flavor and crunchiness. They are also processed into beverages such as beer [7,8,9]. Thus, attention started to be paid to the nutritional profile of this “sea asparagus” [10] a decade ago. Among other properties, it has been found that *Salicornia* members have a high content of polyunsaturated fatty acids (PUFAs) and polyphenols, which are beneficial for human consumption because of their antioxidant properties [7,9]. In halophytes, it is known that these molecules are accumulated to higher concentrations in unstressed conditions, as compared to glycophytes, to cope with salinity-induced oxidative stress [11]. In *S. ramosissima*, the most abundant PUFAs are linolenic acid (omega-3) and linoleic acid (omega-6), which are essential FAs for humans because the human body is not able to synthesize them [12]. Regarding total phenol content, *Salicornia* members [13] record the smallest values in polyphenols compared to other chenopods such as *Halimione* or *Atriplex* [14]. Even so, Ventura et al. [15] found up to 1.2 mg GAE g^−1^ FW in *S. persica*, while the lower limit of other non-halophytic leafy vegetables rated as rich in phenolic compounds is >0.5 mg GAE g^−1^ FW [11]. On the other hand, care should be taken for *Salicornia* ingestion because of its high salt and oxalate content. Salt excess in the human diet is a major risk factor, especially for hypertension, while oxalic acid binds blood calcium by forming crystals of insoluble calcium oxalate, leading to blood hypocalcemia and the formation of renal stones [7]. Members of Chenopodiaceae family are known to contain a high oxalate content [7,16]. They use oxalic acid for the maintenance of the ionic equilibrium, especially for high-capacity calcium regulation [17] and also as a protection mechanism against herbivory, as the ingestion of large amounts of oxalic acid may cause animal death by blood pH disruption [6]. Lastly, the capacity of halophytes for heavy metal resistance and detoxification has been linked to complexation reactions with oxalic acid [18,19,20].

Altogether, *S. ramosissima* may be an alternative cash crop to exploit less arable lands which are unsuitable for conventional crops. However, climate change should be considered as a source of abiotic stress, as it is causing the rise of temperatures and CO_2_ and some associated events such as droughts [21]. Research is needed in order to predict the physiological and biochemical response of *S. ramosissima* and assess its eligibility for breeding programs and human consumption. To date, it is known that *S. ramosissima* has an optimal growth at low salinity (200 mmol L^−1^ NaCl), although it tolerates high salinity (600 mmol L^−1^ NaCl) [22]. Generally, an elevated atmospheric CO_2_ concentration (700 ppm) has a plant-growth-promoting effect in C3 plants, and this includes an improvement in *S. ramosissima* plant physiological performance by increasing net photosynthetic rate (A_N_) and water-use efficiency (iWUE), promoting the accumulation of osmoprotective compounds and the modulation of enzyme antioxidant machinery [23,24]. Finally, we know that short-term, extreme temperature events have a negative impact on *S. ramosissima* fitness, as revealed by lower antioxidant enzyme activity and the decrease in its photosynthetic efficiency [25]. These shifts may lead to variations in *S. ramosissima* chemical composition and, consequently, in its nutritional value. However, research in the nutritional profile of halophytes under abiotic stress is very scarce. To date, it has mostly focused on revealing the salt effect as an approach to their domestication and irrigation with saline water. For example, Maciel et al. [26] revealed that *S. ramosissima* showed higher levels of omega-3 FA when produced in marine aquaponics, compared with wild populations. In parallel, Lima et al. [27] found the greatest amounts of phenolic compounds in *S. ramosissima* shoots when they grew in intermediate levels of salinity (between 110 and 200 mmol L^−1^ NaCl). Nevertheless, nothing is known about the response of FA, polyphenols, and oxalates in *S. ramosissima* under raised temperature and CO_2_, or drought.

Lastly, the use of specific halotolerant plant-growth-promoting bacteria (PGPB) associated with halophytes could be an important practice for crop production under adverse conditions [28,29,30]. Some authors have found that the use of PGPB in *S. bigelovii* resulted in increased shoot growth and seed yield [31,32,33,34,35,36]. In *S. ramosissima*, bacterial inoculation has been applied only to studying seed germination response [37] and plant phytoremediation capacity [38]. From a nutritional perspective, the effect of PGPB has never been assessed in *S. ramosissima*.

Thus, the objective of this work is to gain insight on the aforementioned questions and to elucidate the effect of simultaneous temperature and CO_2_ rise, salinity, and drought, together with PGPB inoculation, on *S. ramosissima* nutritional value, with an emphasis on its content in FA, polyphenols, and oxalates. The coexistence of stressors, despite its logistical complexity, reflect the environmental reality in a more reliable way, as climatic events in nature occur jointly. Based on the bibliography, we hypothesize that high salinity, high temperature, and drought may increase *S. ramosissima* content in FA, phenols, and oxalates to counteract stress. Moreover, we hypothesize that elevated CO_2_ and PGPR inoculation may improve plant fitness and, therefore, cushion nutrient disruption. This knowledge may open new perspectives for the nutritional and economical valorization of *S. ramosissima*.

## 2. Results

### 2.1. Effect of Combined Temperature, CO_2_, Irrigation, and Biofertilization on S. ramosissima Shoots Fatty Acid Content

**Figure 1:**
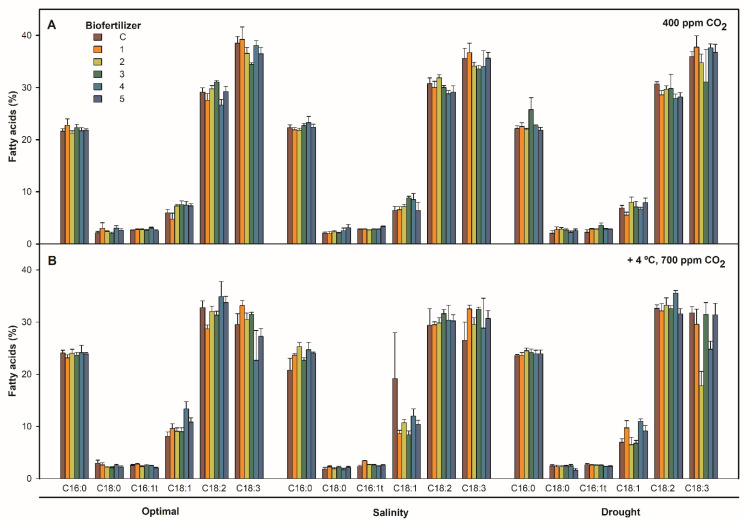
Relative content of FA in *S. ramosissima* shoots, as expressed by percentage, after 30 days under irrigation (optimal 171 mmol L^−1^ NaCl, salinity 510 mmol L^−1^ NaCl, and drought) and inoculation (control non-inoculated—C, and biofertilizers 1 to 5) treatments in (**A**) 25/14 °C 400 ppm CO_2_ chamber and (**B**) 29/18 °C 700 ppm CO_2_ chamber. Values are means ± S.E. (*n* = 5) (GLM: T/CO_2_ × Irrigation × Inoculation, *p* > 0.05).

Data obtained for saturated palmitic (C16:0) and stearic acid (C18:0), monounsaturated chloroplastidial trans-hexadecenoic acid (C16:1t) and oleic acid (C18:1), and polyunsaturated linoleic (C18:2) and linolenic acid (C18:3) were analyzed at the end of the experiment (Figure 1). The FA profile in *S. ramosissima* leaves was dominated by PUFAs (approximately 65%, Table 1), which are represented by linoleic and linolenic acid, followed by saturated FAs (SFAs) (approximately 25%, Table 1) and palmitic and stearic acid. The monounsaturated FAs (MUFAs) group was represented by trans-hexadecenoic and oleic acid (approximately 9%, Table 1). The FA profile was clearly affected by the increment of temperature and CO_2_ (*p* < 0.001, Table 2), as mostly evidenced by an increase in the percentage of C16:0 (6%), C18:1 (40%), and C18:2 (8%) and a decrease in C18:3 (24% on average). This was confirmed by the increase in the 18:2/18:3, PUFA/MUFA, and Omega 6 indexes and the decrease in the Omega 3 index (Table 1). Differences among irrigation treatments were significant only for C18:3 content (*p* < 0.05, Table 2), which was slightly reduced by drought, whereas PGPR inoculation did not produce changes in *S. ramosissima* FA profile (*p* > 0.05, Table 2). The interaction of parameters did not show generalized significant differences on FA profile (*p* > 0.05, Table 2).

On the other hand, our results (Table 1) showed that the edible shoots of *S. ramosissima* had a health-promoting lipid content and that they were altered by temperature and CO_2_ rise. AI and TI values were low and consistent among all treatments (0.2–0.3), slightly increasing at higher temperature and atmospheric CO_2_ (0.2–0.4), as did h/H values (2.5–4). Regarding lipid oxidation, OS values were high at approximately 4400–5200 but dropped with raised temperature and atmospheric CO_2_ to 3200–4700, as did Cox values, which dropped from 11 to 9, approximately. The PI value followed the same pattern, dropping from 70–80 to 60–75.

### 2.2. Effect of Combined Temperature, CO_2_, Irrigation, and Biofertilization on Total Phenol Content in S. ramosissima Shoots

**Figure 2:**
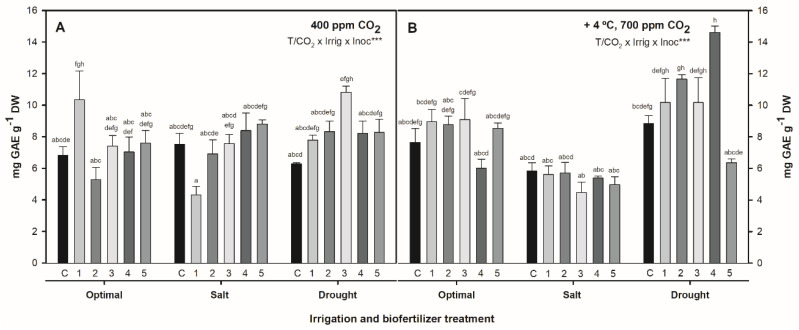
Total phenol content in *S. ramosissima* shoots, as expressed by mg GAE g^−1^ DW, after 30 days under irrigation (optimal 171 mmol L^−1^ NaCl, salinity 510 mmol L^−1^ NaCl, and drought) and inoculation (control non-inoculated—C, and biofertilizers 1 to 5) treatments in (**A**) 25/14 °C 400 ppm CO_2_ chamber and (**B**) 29/18 °C 700 ppm CO_2_ chamber. Values are means ± S.E. (*n* = 3). Different letters indicate means that are significantly different from each other (GLM: T/CO_2_ × Irrigation × Inoculation, *p* < 0.001). “T/CO_2_ × Irrig × Inoc” in the upper right corner of the panels indicates triple interaction significant effects (*p* *** < 0.001).

Total phenol content at the end of the experiment in *S. ramosissima* shoots is represented in Figure 2. In non-biofertilized plants, concentration of total phenols ranged from 5 to 9 mg GAE g^−1^ DW. Increases in temperature and CO_2_ levels did not cause significant differences in phenol content (*p* > 0.05). At optimal temperature/CO_2_, salinity or drought also did not cause significant differences in phenol content (*p* > 0.05). However, at +4 °C and 700 ppm atmospheric CO_2_, saline irrigation induced a decrease in phenol content (*p* < 0.05) in *S. ramosissima* leaves, whereas drought induced a slight increase. The combination of all the parameters studied, including biofertilization, had a significant effect on the phenol content of *S. ramosissima* leaves (Table 2, GLM: T/CO_2_ × Irrigation × Inoculation, *p* < 0.001). Biofertilization with PGPR produced a phenol increase under drought in both optimal and raised T/CO_2_. In particular, a very high phenol content was detected in *S. ramosissima* plants inoculated with rhizobia inoculum number 4, reaching 15 mg GAE g^−1^ shoot DW, a 65% increase over its respective non-inoculated control.

### 2.3. Effect of Combined Temperature, CO_2_, Irrigation, and Biofertilization on Oxalate Concentration in S. ramosissima Shoots

**Figure 3:**
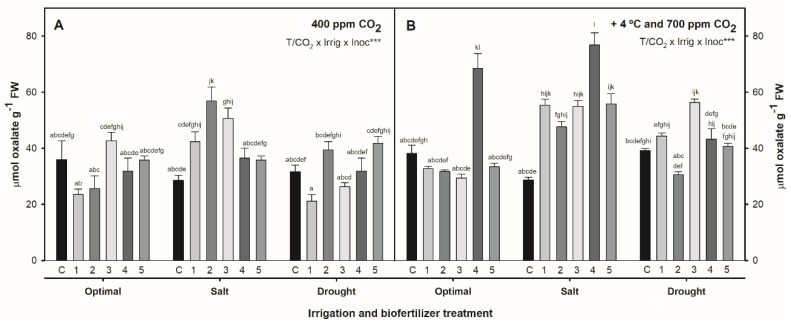
Oxalate content in *S. ramosissima* shoots, as expressed by µmol oxalate g^−1^ FW, after 30 days under irrigation (optimal 171 mmol L^−1^ NaCl, salinity 510 mmol L^−1^ NaCl, and drought) and inoculation (control non-inoculated—C, and biofertilizers 1 to 5) treatments in (**A**) 25/14 °C 400 ppm CO_2_ chamber and (**B**) 29/18 °C 700 ppm CO_2_ chamber. Values are means ± S.E. (*n* = 3). Different letters indicate means that are significantly different from each other (GLM: T/CO_2_ × Irrigation × Inoculation, *p* < 0.001). “T/CO_2_ × Irrig × Inoc” in the upper right corner of the panels indicates triple interaction significant effects (*p* *** < 0.001).

Oxalic acid concentration in *S. ramosissima* leaves at the end of the experiment is shown in Figure 3. In non-biofertilized plants, oxalic acid ranged from 25 to 40 µmol g^−1^ FW. Temperature/CO_2_ rise and drought did not cause significant differences in oxalate content (*p* > 0.05). However, saline irrigation (510 mmol L^−1^ NaCl) caused a decrease in oxalate content, which was more pronounced at +4 °C and 700 ppm CO_2_ (*p* < 0.05). The combination of all the parameters studied, including biofertilization, had a significant effect on the oxalate concentration of *S. ramosissima* leaves (Table 2, GLM: T/CO_2_ × Irrigation × Inoculation, *p* < 0.001). Rhizobacteria biofertilization produced a significant increase in oxalate levels under salinity, being more evident at +4 °C and 700 ppm CO_2_. Plants inoculated with the 5 PGPR consortia showed significantly higher oxalate content than the non-inoculated plants (*p* < 0.05), especially consortium number 4, which increased the leaf oxalate content up to 2.6× at 515 mmol L^−1^ NaCl and 1.7× at optimal 171 mmol L^−1^ NaCl compared to non-inoculated *S. ramosissima*.

## 3. Discussion

We analyzed the combined effect of increased temperature and CO_2_, salt and drought treatments, and PGPR inoculation for 30 days on the *S. ramosissima* shoot nutrient content. As an edible cash crop, the contents of FAs, oxalate, and phenolic compounds in *S. ramosissima* shoots are very important from a nutritional point of view but also from a physiological perspective as part of a complex mechanism for abiotic stress tolerance.

FA profile, besides its nutritional relevance, has been suggested as a useful biomarker for abiotic stress in halophytes species, such as *Spartina maritima*, *Spartina patens*, *Halimione portulacoides,* and *Sarcocornia fruticosa*, and their levels are highly related to the photosynthetic functioning of these species [39,40]. We found that *S. ramosissima* leaves were dominated by PUFAs (especially by linolenic and linoleic acid), followed by SFAs (mostly palmitic acid), in agreement with the results obtained for other *Salicornia* species such as *S. europaea* [41] and *S. bigelovii* [42]. On the other hand, Isca et al. [43], who studied for the first time the lipid profile of *S. ramosissima*, found a greater proportion of SFAs rather than PUFAs. In agreement with these results are also the data obtained by Radwan et al. [44] for *S. fruticosa*. Such saturated FA profiles are common in halophytic plants and may be related to salt tolerance mechanisms [9], probably through a decrease in membrane permeability to NaCl. However, Elsebaie et al. [45] reported oleic MUFA as the most abundant in *S. fruticosa* (more than 50% of FA). Altogether, it seems that the differences observed in the fatty acid composition in *Salicornia* are mainly related to the species and morphological zone of the plant under study, besides environmental conditions [43]. In our work, an increase in temperature by 4 ºC and atmospheric CO_2_ to 700 ppm caused a clear impact on the FA profile of *S. ramosissima* shoots, leading to the loss of unsaturation degree, as shown by the increase in the 18:2/18:3, PUFA/MUFA, and Omega 6 indexes and the decrease in the Omega 3 index. The same response was observed by Pérez-Romero et al. [23]. According to several reports, lipids may be involved in the PSII protection in the presence of salt stress [46,47,48]. In photosynthetic tissues, linolenic acid is associated with the synthesis of galactolipids monogalactosyldiacylglycerol (MGDG) and digalactosyldiacylglycerol (DGDG), which are fundamental for the correct function of photosynthesis [49]. Moreover, a direct action of ROS production during stress exposition has been described in the literature [50,51], which may lead to PUFA degradation by direct action in double bonds. These mechanisms may explain the decrease that we observed in *S. ramosissima* relative content of C18:3 upon a temperature and CO_2_ rise. However, according to Duarte et al. [48], there was no correlation between the decrease in C18:3 and the photosynthetic apparatus performance in the halophyte *Aster tripolium*. We expected more evident changes in the FA profile of *S. ramosissima* under salinity and drought, as observed by Maciel et al. [26] in *S. ramosissima* grown in marine aquaponics, which had higher levels of n-3 FA compared with the wild populations. However, compared to the effect of temperature/CO_2_, we did not find significant differences in *S. ramosissima* FA profile with salinity or drought, and neither did other authors [15,43]. Trans-hexadecenoic acid (16:1t), a fatty acid that is present exclusively in the chloroplast [52], is generated by a membrane-bound desaturase acting on C16:0, which is esterified to phosphatidylglycerol (PG), which is the only phospholipid present in thylakoids. The decrease in the concentration of this fatty acid observed under drought indicates a reduction in the fluidity of the chloroplast membrane [53]. FA quality indexes showed that the increase in temperature and CO_2_ predicted for the future atmosphere decreased the quality of FA, in terms of oxidative stability. That is, they are more prone to oxidation, and in terms of human health, they have a lower protective potential for the cardiovascular system [39].

Regarding the changes in phenolic compounds, only a few studies have analyzed total phenol content in *S. ramosissima*. The values here reported, from 5 to 9 mg GAE g^−1^ DW (4 to 14 mg GAE g^−1^ DW in inoculated plants), were similar to the values found by other authors for pot-grown *S. ramosissima* (9 to 13 mg GAE g^−1^ DW [27]). These concentrations seem to be greater for wild *S. ramosissima* populations. For example, Barreira et al. [9] detected up to 33 mg GAE g^−1^ DW, while Antunes et al. [54] found up to 40 mg GAE g^−1^ DW and 24 mg GAE g^−1^ DW for plants harvested in July and May, respectively. Duarte et al. [55] also found higher phenolic values in several halophytes during spring and summer, suggesting that it may be a strategy to cope with oxidative stress due to high light intensities and increased salinity during the summer. Moreover, some flavonoid production is boosted under high irradiation to reflect excessive solar energy. Our results are in agreement for the reported range of phenol content for *Salicornia* species, that is, from 1 to 50 mg GAE g^−1^ DW (*S. herbacea* [56,57,58], *S. bigelovii* [15] or *S. europaea* [14]). It seems logical that phenols may increase under salt stress conditions in order to help the plants to cope with salinity, as has been demonstrated by Wang et al. [59] for tobacco and also in other species such as *Hyssopus officinalis* [60] and *Apera spica-venti* [61]. In halophytes, several authors have reported the same pattern, as they observed up to a 1.5× increase in total phenol content after irrigation with seawater [15,62,63]. In this study, we observed a slight but significant increase in phenol content as a response to saline irrigation in *S. ramosissima*. Despite the fact that this result appears to indicate an adaptation response to salinity, other authors observed the opposite effect, and Lima et al. [27] concluded that *S. ramosissima* cultivated with intermediate levels of salinity (110 and 200 mmol L^−1^ NaCl) showed higher amounts of phenolic compounds, while Kang et al. [57] reported higher levels of polyphenols in freshwater-cultivated *S. herbacea* than naturally-grown plants. The halophyte *Haloxylon stocksii* also showed a decrease in total phenol content after watering with media containing either 100 or 300 mmol L^−1^ NaCl, compared to control plants [64]. Therefore, it seems that there is not a common pattern of response to salinity conditions in different species of halophyte plants. This could be due to the fact that different systems may be used in each species in order to adapt to with salt stress. For example, our results showed a different response of phenol content to saline irrigation at ambient CO_2_ and at elevated CO_2_. The decrease in phenol content at a higher atmospheric CO_2_ concentration may probably be driven by the fitness improvement mediated by CO_2_ in *S. ramosissima* [24,65]. Contrary to that, in non-halophyte species, increased temperatures and/or CO_2_ clearly induced the accumulation of total phenols in leaves as a defense mechanism, such as in *Vallisneria spiralis* [66], *Alternanthera sessilis* [67], chickpea [68], and garlic [69]. Production of these antioxidant compounds can be induced by other stress abiotic conditions rather than salinity, such as UV-C radiation, floods, or drought [70]. Accordingly, in our experiment, elevated temperature and drought increased phenol content in *S. ramosissima* shoots, probably as an antioxidant defense mechanism.

Contrary to FA and phenols, low levels of oxalates are a desirable trait for human consumption. However, it should not be forgotten that oxalic acid is a basic plant mineral regulator. In rice, it has been suggested that oxalate accumulation has a genetic basis and does not depend on CO_2_ and temperature conditions [71]. In *Rumex obtusifolius,* it has been observed that 600 ppm atmospheric CO_2_ lowered the concentration of oxalate in leaves, compared to ambient CO_2_ [72]. In our work, increases in temperature and atmospheric CO_2_ resulted in lower levels of oxalate under saline irrigation. This effect may be due to complexation of free oxalic acid to maintain ionic equilibrium. In the same line, Khan et al. [73] found that non-saline controls of the halophyte *Cressa cretica* showed higher oxalate concentrations and that it was also related to plant population density. Accordingly, *S. europaea* showed a reduction of 80% of oxalate content in response to increasing NaCl concentration. Nevertheless, in a similar way to what we observed for phenolic compound content, there is not a consistent pattern in response to salinity for the levels of oxalates. Other authors have found out that the increase in salinity in the growth medium of halophytes resulted in higher oxalate content [64,74,75].

Finally, we found that the effect of bacterial inoculation in *S. ramosissima* depended greatly on the bacterial strains that were employed, as reported previously by other authors [76]. Moreover, we observed that bacterial properties are more important than their origin, as the consortia with more interesting effects in our experiment were number 3 and 4, which were originally isolated from the rhizosphere of halophytes *Spartina maritima* and *Atriplex portulacoides*, while consortium number 5, which was isolated from *S. ramosissima*, did not produce relevant responses. High and middle marsh area colonized by *A. portulacoides* is more prone to higher salinities and lower water availability, so it may be expected that bacteria from these sites might be adapted to counteract these effects. Other authors have proven that bacterial inoculation reduces the negative effects of salinity stress by means of phenol accumulation [77]. The rationale behind this pattern could be related to the host defense response, which is linked to the activation of secondary pathways associated with the onset of induced resistance, including the oxidation and polymerization of pre-existing phenols and the synthesis of new phenolic compounds via the activation of the phenylpropanoid pathway [78]. However, in our experiment, phenol accumulation after bacterial inoculation was evidenced only in plants subjected to drought. In contrast with other authors, we did not observe phenol accumulation at saline irrigation. Rueda-Puente et al. [35] observed that *S. bigelovii* seedlings at 250 mmol L^−1^ NaCl inoculated with *K. pneumoniae* increased their phenolic content by 19% compared to non-inoculated controls. Their results were in agreement with those of Dong et al. [79], who additionally reported that *K. pneumoniae* established endophytic populations in *S. bigelovii*. Then, perhaps bacteria that colonize inner plant tissues induce a greater host defense response and, consequently, a greater phenol accumulation. In this work, the bacteria used are salt-tolerant rhizobacteria, which naturally grow in soil and root surfaces at 510 mmol l^−1^ NaCl. However, the loss of soil moisture may be a critical stress factor that induces some bacteria to colonize plant roots after seeking more appropriate living conditions. On the other hand, oxalate content increased with saline irrigation of *S. ramosissima*. It may be related to their ability to counteract salt stress and promote plant growth, which involves plant biomass increment and ion uptake and, therefore, the need to support the ionic homeostasis machinery. Altogether, consortia 3 and 4 greatly increased phenol and oxalate accumulation in response to saline irrigation and drought. Biofertilizer 4 was the only one which had an ACC deaminase producer strain (HPJ2), an important characteristic to aid plants to face abiotic stress [80], while biofertilizer 3 relied on plant-growth-promoting *Bacillus* strains, which are known to be very tolerant to abiotic stress [81]. Taking into account previous reports, it would be advisable to verify if there is inner tissue colonization by inoculated bacteria [82]. Lastly, contrary to the effect observed in phenol and oxalate content, biofertilization did not cause significant changes in FA profile, nor did it lessen the effect of raised temperature and CO_2_. In short, according to our results, biofertilization may promote the accumulation of phenols in the leaves of *S. ramosissima* in a climate change context, while may not alter FA profile. Nonetheless, PGPR inoculation may cause the accumulation of oxalate if salinized soils or irrigation water are used for plant breeding, which should be taken into consideration.

## 4. Materials and Methods

### 4.1. Plant Material

*S. ramosissima* seeds were harvested from individual plants (*n* = 30) that were randomly selected from a well-established population located in Odiel salt marshes (37°15′ N, 6°58′ O; SW Spain) and immediately transported to the laboratory and stored at 4 °C in the dark for 3 months. After storage, period seeds were surface-disinfected by immersion and vigorous shaking in 5% sodium hypochlorite (*v*/*v*) for 1 min, followed by several rinses with sterile distilled water. The sterilized seeds were then placed in Petri dishes in a germinator (ASL Aparatos Científicos M-92004, Madrid, Spain) and subjected to a day/night regime of 16 h of light (photon flux rate, 400–700 nm, 35 μmolm^−2^ s^−1^) at 25 °C and 8 h of darkness at 12 °C for 15 days. The germinated seedlings were planted in 0.25 L individual plastic plots containing a mixture of an organic commercial substrate (Gramoflor GmbH and Co. KG., Vechta, Germany) and sand (2:1) that was previously disinfected, and placed in controlled environment chambers (Aralab/Fiberoclima 18.000 EH, Lisbon, Portugal), which were programmed with an alternate daily regime of 16 h/8 h and 25/14 °C (light/darkness) with a light intensity of 300 μmol m^−2^ s^−1^ and 50 ± 5% relative humidity and watered with a 171 mmol L^−1^ NaCl saline solution. The plants were kept under these conditions until the onset of the experiment.

### 4.2. Experimental Design and Treatments

**Figure 4:**
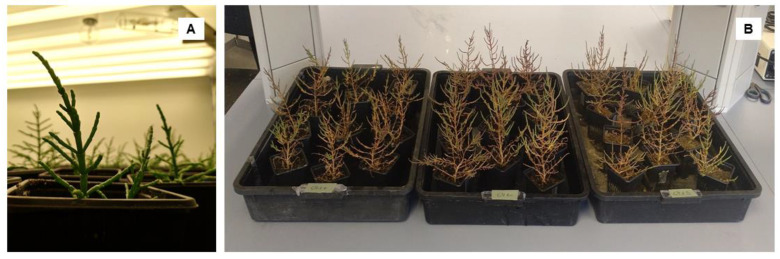
Pictures of *Salicornia ramosissima* (**A**) during the experiment inside the controlled environment chamber and (**B**) at the end of the experiment before sample processing (trays shown were grown in the high temperature and CO_2_ chamber, with saline, optimal, and deficient irrigation, and inoculated with bacterial consortium number 4).

After one month of seedling growth, when the plants had a mean height of 11 cm, the pots were randomly subjected to 36 different treatments for 30 days (*n* = 10 per treatment, 360 plants in total) (see overview in Table 3): 2 combinations of CO_2_–temperature (400 ppm CO_2_ at 25/14 °C (16/8 h) and 700 ppm CO_2_ at 29/18 °C (16/8 h)); 3 irrigation regimens (optimal 171 mmol L^−1^ NaCl, salt stress 510 mmol L^−1^ NaCl, and drought stress); and 6 bacterial inoculation treatments (5 different rhizobacteria consortia and 1 non-inoculated control) (Figure 4). Optimal and stress salt concentrations were chosen based on previous experiences of assessing salt tolerance of *S. ramosissima* [25,37]. The atmospheric CO_2_ concentrations in the chambers were continuously recorded by CO_2_ sensors (Aralab, Lisbon, Portugal) and maintained by supplying pure CO_2_ from a compressed gas cylinder (Air Liquide, B50 35 K). Rhizobacterial inoculation was carried out the day after environmental treatments (salinity, CO_2_, and temperature). After the establishment of each treatment, the pots were watered daily with 100 mL of 20% strength Hoagland’s solution. For the establishment of optimal and salinity irrigation treatments, 2 groups of plants were watered with the specific saline concentration (i.e., 171 or 510 mmol L^−1^ NaCl) to field capacity at the beginning of the experiment. The pots were kept under well-watered conditions throughout the experimental period by placing pots in individual plastic trays containing the appropriate NaCl solutions to a depth of 1 cm. To avoid changes in the NaCl concentration caused by water evaporation, the levels in the trays were continuously monitored throughout the experimental period. Drought conditions were established by withholding water until the soil water content dropped by 40% by comparing the mass of the pots with the corresponding mass at field capacity, which occurred on day 4. This group was kept under water-stressed conditions throughout the entire experimental period by adding the amount of specific optimal saline solution (171 mmol L^−1^ NaCl) that they lost during the day. Additionally, to avoid an increase in soil salinity concentration due to drought/saline irrigation daily events, periodic soil electrical conductivity measurements were carried out.

### 4.3. Rhizobacteria Selection and Inoculation

Five bacterial biofertilizers were used for the experiments carried out with PGPB. These bacteria had been previously tested in up to eight crops [76], as well as in a germination assay with *S. ramosissima* seeds [37]. They were composed of rhizobacteria originally isolated from the rhizospheres of 5 different halophytes, commonly inhabiting salt marshes in southwestern Spain (Tinto, 37°13′ N 6°53′ W; Odiel, 37°10′35.2″ N 6°55′59.2″ W; and Piedras, 37°16′09.1″ N 7°09′36.4″ W, river estuaries). The rhizobacteria that composed the five different biofertilizers were chosen because they showed potential for promoting plant growth, as well as salt tolerance up to 2 mol L^−1^ NaCl (see Table 4). To prepare the bacterial suspensions for inoculation, the strains were grown separately in 250 mL Erlenmeyer flasks containing 50 mL of liquid TSB (Tryptone Soya Broth) medium and incubated on a rotary shaker for 18–24 h at 28 °C. The cultures were then centrifuged in 50 mL Falcon tubes at 7000 rpm (6300× *g*) for 5 min, and the supernatant was discarded. The pellets were washed twice with sterile tap water (by resuspension and centrifugation) and finally resuspended in tap water to reach an OD_600_ of approximately 1.0 in order to produce a uniform bacterial concentration of all strains. The individual bacteria suspensions were mixed to produce the five final inoculant suspensions as follows: strains SDT3, SDT13, and SDT14 were mixed to obtain Biofertilizer 1; strains RA1, RA15, and RA18 were mixed to obtain Biofertilizer 2; strains SMT38, SMT48, and SMT51 were mixed to obtain Biofertilizer 3; strains HPJ2, HPJ15, and HPJ50 were mixed to obtain Biofertilizer 4; and strains SRT1, SRT8, and SRT15 were mixed to generate Biofertilizer 5. For plant inoculation, every 0.25 L pot was watered with 15 mL of the inoculant suspensions to obtain a final bacteria concentration of 10^6^ CFU/mL (estimating that a suspension of OD_600_ of 1 corresponds to approximately 10^8^ CFU/mL).

### 4.4. Fatty Acid Profiling of S. ramosissima Shoots

At the end of the experiment, between 200 and 300 mg of aerial branches (*n* = 5 per treatment) were collected in liquid nitrogen. Leaf fatty acid composition was determined via direct acidic *trans*-esterification of pre-weighted leaf portions, as per [48]. Freshly prepared 3 mL methanol–sulfuric acid solution (97.5:2.5 *v*/*v*) was added to the samples, and they were heated at 70 °C for 60 min, at which point, a methylation reaction occurs. Fatty acid methyl esters (FAMEs) were then recovered via extraction by adding a mixture of 3 mL petroleum ether and 2 mL ultrapure water, vortexing, and centrifuging the samples at 4000 rpm for 5 min. The upper phase was collected, and the organic solvent was evaporated in a dry bath at a constant temperature of 30 °C under a continuous N_2_ flow. FAMEs were resuspended in 30 μL of hexane, and 1 μL was injected into a gas chromatograph (3900 Gas Chromatograph, Varian), which was equipped with a hydrogen flame-ionization detector set at 300 °C. The temperature of the injector was set to 270 °C, with a split ratio of 50. The fused-silica capillary column (50 m × 0.25 mm i.d.; WCOT Fused Silica, CP-Sil 88 for FAME; Varian) was maintained at a constant N_2_ flow of 2.0 mL min^−1^, and the oven was set to 190 °C. Identification of different FA was achieved via a comparison of retention times with analytical standards (Sigma-Aldrich, USA), and chromatograms were analyzed via the peak surface method by using Galaxy software. Heptadecanoic acid (C17:0) was used as an internal standard. The relative FA composition of each sample was characterized by the mean percentage and standard variation in individual FA, as well as by FA classes based on saturated fatty acids (SFA, fatty acids without double bonds), monounsaturated fatty acids (MUFA, fatty acids with a single double bond), and polyunsaturated fatty acids (PUFA, fatty acids with 2 or more double bonds):

SFA = [C14:0] + [C16:0] + [C18:0](1)

MUFA = [16:1t] + [18:1](2)

PUFA = [C18:2] + [C18:3](3)

Furthermore, to assess the nutritional quality of the lipids present in the leaves of *S. ramosissima* and considering their protective role in cardiac diseases, the atherogenicity (AI) and thrombogenicity (TI) indexes, oxidizability (Cox), oxidative susceptibility (OS), hypocholesterolemic/hypercholesterolemic index (h/H), and peroxidizability index (PI) were calculated according to the following equations [39]:

(4) $$AI=\frac{\left(C16:0+C18:0\right)}{MUFAs+PUFAs_{n3}+PUFAs_{n6}}$$

(5) $$TI=\frac{\left(C16:\;0+C18:\;0\right)}{(0.5\times MUFAs+0.5\times PUFAs_{n6}+3\times PUFAs_{n3}+\left(\frac{PUFAs_{n3}}{PUFAs_{n6}}\right)}$$

(6) $$C_{ox}=\frac{\left(C18:1+10.3\times C18:2+21.6\times C18:\;3\right)}{100}$$

(7) OS=MUFA+45×C18: 2+100×C18: 3

(8) $$\frac{h}{H}=\frac{\left(C18:\;1+C18:\;2+C18:\;3\right)}{C16:0}$$

(9) PI=(C16: 1t+C18: 1)×0.025+C18: 2+2×C18: 3

The smaller the AI and TI values, the greater the protective potential for the cardiovascular system. While Cox values should be low, indicating that FAs are less prone to oxidation, OS should be as high as possible. This indicated the effects of specific FA on cholesterol metabolism. This index is of fundamental importance because the h reduces the low-density lipoprotein cholesterol, also known as bad cholesterol, whereas the H increases it. The peroxidizability index (PI) is used to assess the stability of PUFAs and their capacity to be protected from possible oxidation processes. High h/H and PI index values are considered more beneficial for human health [39].

### 4.5. Determination of Total Phenolic Content in S. ramosissima Shoots

At the end of the experiment, aerial branches (*n* = 5 per treatment) were collected, freeze-dried, and finely ground with a grinder. Pulverized samples were stored at room temperature in a vacuum container until processing. A pool from the five plants was prepared, and three replicates per treatment were obtained. The total content of phenolic compounds was assessed using the Folin–Ciocalteu method. Briefly, *S. ramosissima* samples were diluted to 10 mg/mL in distilled water, followed by sonication for 30 min. Samples were then centrifuged at 3000× *g* for 10 min and diluted to 1 mg/mL in water. Then, 30 μL of each sample (1 mg/mL) or standard (gallic acid) were mixed in triplicates with 30 μL of Folin–Ciocalteu reagent (1:10 dilution in water) in a 96-well plate and incubated at room temperature for 5 min. Finally, 240 μL of 5% sodium carbonate (*w*/*v*) was added to each well, and the plate was incubated for 1.5 h at 30 °C. Absorbance was measured at 760 nm by using a CLARIOstar spectrophotometer (BMG Labtech, Germany). Total phenolic content was calculated by using a gallic acid calibration curve within a range of 0–100 μg/mL. Results were expressed as mg gallic acid equivalents (GAE) g^−1^ of dry material weight (DW).

### 4.6. Determination of Oxalate Content in S. ramosissima Shoots

At the end of the experiment, *S. ramosissima* fresh aerial branches (*n* = 3 per treatment) were ground with mortar and pestle with liquid nitrogen and stored at −80 °C until used. In total, 500 µL of ice-cold Tris-HCl 0.1 mol L^−1^ pH 7.6 buffer was added to 50 mg of frozen tissue. The tissue was homogenized in ice by using a pellet homogenizer. The homogenate was centrifuged for 5 min at 13,000 rpm at 4 °C. The supernatant was diluted by 1:10 in Tris-HCl 0.1 mol L^−1^ pH 7.6 buffer, and 10 µL of this dilution was used for oxalic acid determination. Oxalate content was determined by using an oxalate assay kit (Sigma-Aldrich, USA, reference MAK315) according to the manufacturer’s instructions.

### 4.7. Statistical Analysis

Statistical analysis was carried out by using Statistica v. 10.0 (Statsoft Inc.). Data were first tested for normality with the Kolmogorov–Smirnov test and for homogeneity of variance with the Brown–Forsythe test. Then, generalized linear models (GLMs) were used to analyze the interactive effects of temperature/CO_2_, irrigation, and biofertilizers (as categorical factors) on the content of FA, total phenols, and oxalates in *S. ramosissima* shoots (as dependent variables). The Tukey test was applied to establish the significance between treatments (*p* < 0.05) [86].

## 5. Conclusions

In this work, we demonstrated that increased temperature and atmospheric CO_2_, together with salinity and drought conditions, led to important changes in the FA, phenol, and oxalate content of *S. ramosissima*. The FA profile was affected by increments in temperature and CO_2_ such as the ones that are predicted for the future climate change scenario, which may lead to a loss of FA unsaturations and an impairment of their quality in terms of oxidative stability and protective potential for cardiovascular human health. The total phenolic compound content decreased under salinity and increased under drought, but that was only under high temperature and CO_2_. Oxalate content decreased with salinity, and the change was more pronounced at higher temperature and CO_2_. Thus, the future climate scenario may alter the *S. ramosissima* lipid profile toward the loss of FA unsaturations and may lead to oxalate and phenol contents that are more sensitive to salinity and drought.

The effect of inoculation with PGPR depended on the strain that was used and their characteristics. Some strains induced the accumulation of phenols in *S. ramosissima* leaves at higher temperature and CO_2_ and did not alter FA profile but also led to an accumulation of oxalate in plants under salt stress. This work demonstrates the importance of tacking combined approaches in the study of different stressors, which is opposite to the typical control/stress approach, because this situation occurs as these conditions merge together in the environment. This study may hint to which changes in the content of important metabolites for human health may take place in *S. ramosissima* in the future climate change scenario and how the nutritional value of this edible crop may change according to the interaction of the different conditions/parameters that were studied in this work.

## Figures and Tables

**Table 1:** Fatty acids for *S. ramosissima* shoots after 30 days of the experiment: saturated FA (SFA), monounsaturated FA (MUFAs), polyunsaturated FA (PUFA), and indexes of lipid nutritional quality: oxidizability (Cox), oxidative susceptibility (OS), hypocholesterolemic/hypercholesterolemic (h/H), peroxidizability (PI), atherogenic index (AI), thrombogenic index (TI), and omega 3 and omega 6. Values are means ± S.E. (*n* = 5).

**Irrigation**	**Optimal (171 mmol NaCl L^−1^)**											
**T and CO_2_**	**400 ppm**						**+4 °C, 700 ppm**					
**Inoculation**	**C**	**1**	**2**	**3**	**4**	**5**	**C**	**1**	**2**	**3**	**4**	**5**
SFA	23.8 ± 0.5	25.7 ± 2.3	23.7 ± 0.4	24.3 ± 0.5	24.7 ± 0.3	24.4 ± 0.4	27.0 ± 0.3	25.8 ± 0.6	26.1 ± 0.6	25.7 ± 0.4	26.6 ± 1.6	26.1 ± 0.2
MUFA	8.6 ± 0.7	7.5 ± 1.1	10.0 ± 0.4	10.2 ± 0.6	10.5 ± 0.9	9.9 ± 0.3	10.6 ± 0.9	12.4 ± 1.0	11.4 ± 0.7	11.5 ± 0.9	15.9 ± 1.5	12.9 ± 0.7
PUFA	67.7 ± 0.7	66.8 ± 2.7	66.3 ± 0.6	65.5 ± 0.4	64.7 ± 0.8	65.7 ± 0.6	62.3 ± 1.2	61.8 ± 1.4	62.5 ± 1.0	62.8 ± 0.8	57.5 ± 3.0	61.0 ± 0.6
PUFA/SFA	2.9 ± 0.1	2.7 ± 0.3	2.8 ± 0.1	2.7 ± 0.1	2.6 ± 0.0	2.7 ± 0.1	2.3 ± 0.1	2.4 ± 0.1	2.4 ± 0.1	2.4 ± 0.1	2.2 ± 0.2	2.3 ± 0.0
18:2/18:3	0.8 ± 0.04	0.7 ± 0.05	0.8 ± 0.04	0.9 ± 0.01	0.7 ± 0.04	0.8 ± 0.05	1.14 ± 0.12	0.87 ± 0.03	1.06 ± 0.07	1.00 ± 0.03	1.10 ± 0.06	1.27 ± 0.13
Cox	11.4 ± 0.2	11.4 ± 0.5	11.0 ± 0.2	10.7 ± 0.1	11.0 ± 0.1	10.9 ± 0.2	9.8 ± 0.3	10.2 ± 0.2	10.0 ± 0.2	10.1 ± 0.1	8.6 ± 0.9	9.5 ± 0.2
OS	5171.4 ± 100.5	5170.4 ± 245.0	5005.4 ± 87.3	4853.7 ± 28.6	5017.9 ± 70.4	4972.5 ± 89.7	4441.5 ± 158.5	4618.9 ± 113.0	4499.2 ± 105.0	4566.6 ± 47.4	3852.1 ± 444.8	4256.0 ± 106.2
h/H	3.4 ± 0.1	3.2 ± 0.2	3.5 ± 0.1	3.3 ± 0.1	3.3 ± 0.1	3.4 ± 0.1	2.9 ± 0.1	3.1 ± 0.1	3.0 ± 0.1	3.0 ± 0.1	3.0 ± 0.2	3.0 ± 0.0
PI	83.3 ± 2.1	83.7 ± 4.2	80.6 ± 1.9	76.8 ± 0.5	83.1 ± 1.6	80.2 ± 2.1	67.9 ± 3.3	75.1 ± 1.6	70.0 ± 2.3	71.9 ± 0.5	59.6 ± 8.8	65.4 ± 2.4
AI	0.30 ± 0.01	0.35 ± 0.05	0.31 ± 0.01	0.30 ± 0.01	0.30 ± 0.01	0.30 ± 0.01	0.37 ± 0.01	0.35 ± 0.01	0.35 ± 0.01	0.35 ± 0.01	0.36 ± 0.03	0.35 ± 0.00
TI	0.2 ± 0.01	0.19 ± 0.03	0.18 ± 0.01	0.19 ± 0.00	0.18 ± 0.00	0.2 ± 0.01	0.25 ± 0.01	0.21 ± 0.01	0.23 ± 0.01	0.22 ± 0.00	0.38 ± 0.14	0.25 ± 0.01
Omega 3	38.5 ± 1.3	39.2 ± 2.4	36.6 ± 1.1	34.5 ± 0.3	38.1 ± 0.9	36.5 ± 1.3	29.6 ± 2.0	33.2 ± 1.0	30.5 ± 1.3	31.5 ± 0.4	22.7 ± 5.7	27.3 ± 1.5
Omega 6	29.1 ± 0.8	27.5 ± 1.3	29.7 ± 0.6	31.1 ± 0.3	26.7 ± 1.0	29.2 ± 1.0	32.8 ± 1.3	28.7 ± 0.7	32.0 ± 1.0	31.3 ± 0.8	34.9 ± 2.9	33.7 ± 1.2
**Irrigation**	**Salinity (510 mmol NaCl L^−1^)**											
**T and CO_2_**	**400 ppm**						**+4 °C, 700 ppm**					
**Inoculation**	**C**	**1**	**2**	**3**	**4**	**5**	**C**	**1**	**2**	**3**	**4**	**5**
SFA	24.4 ± 0.6	23.9 ± 0.4	24.1 ± 0.4	24.8 ± 0.3	25.8 ± 1.6	25.5 ± 0.8	22.6 ± 2.5	25.9 ± 0.4	27.2 ± 0.9	24.9 ± 0.5	26.4 ± 0.2	26.1 ± 0.3
MUFA	9.2 ± 0.8	9.4 ± 0.5	9.9 ± 0.5	11.7 ± 0.3	11.3 ± 1.1	9.7 ± 1.7	21.5 ± 8.5	12.1 ± 0.5	13.4 ± 1.4	11.1 ± 0.8	14.4 ± 1.5	13.0 ± 1.0
PUFA	66.4 ± 1.3	66.7 ± 0.8	66.0 ± 0.7	63.6 ± 0.2	62.8 ± 2.6	64.8 ± 1.9	55.9 ± 6.0	62.0 ± 0.9	59.4 ± 2.3	64.1 ± 1.2	59.2 ± 1.6	60.9 ± 0.8
PUFA/SFA	2.7 ± 0.1	2.8 ± 0.1	2.7 ± 0.1	2.6 ± 0.0	2.5 ± 0.2	2.6 ± 0.1	2.5 ± 0.1	2.4 ± 0.1	2.2 ± 0.1	2.6 ± 0.1	2.2 ± 0.1	2.3 ± 0.0
18:2/18:3	0.9 ± 0.1	0.8 ± 0.08	0.9 ± 0.03	0.9 ± 0.02	0.9 ± 0.10	0.8 ± 0.03	1.14 ± 0.10	0.91 ± 0.03	1.05 ± 0.11	0.99 ± 0.06	1.07 ± 0.08	0.99 ± 0.05
Cox	10.9 ± 0.3	11.1 ± 0.3	10.7 ± 0.1	10.4 ± 0.1	10.4 ± 0.6	10.8 ± 0.3	8.9 ± 0.9	10.1 ± 0.2	9.6 ± 0.5	10.3 ± 0.3	9.5 ± 0.4	9.8 ± 0.1
OS	4955.5 ± 157.5	5028.9 ± 131.9	4855.1 ± 69.3	4720.1 ± 39.4	4711.4 ± 281.8	4884.6 ± 138.0	3992.4 ± 444.1	4591.0 ± 73.6	4310.4 ± 236.7	4677.5 ± 140.1	4263.3 ± 171.3	4441.2 ± 44.8
h/H	3.3 ± 0.1	3.4 ± 0.1	3.4 ± 0.1	3.2 ± 0.1	3.1 ± 0.2	3.2 ± 0.1	4.0 ± 0.8	3.0 ± 0.1	2.8 ± 0.1	3.2 ± 0.1	2.9 ± 0.1	3.0 ± 0.1
PI	78.4 ± 3.1	80.5 ± 3.0	76.1 ± 1.2	75.9 ± 1.1	76.3 ± 5.1	78.2 ± 1.8	66.3 ± 4.3	74.0 ± 1.1	69.1 ± 3.8	73.6 ± 2.6	68.6 ± 2.5	71.1 ± 1.1
AI	0.32 ± 0.01	0.3 ± 0.01	0.3 ± 0.01	0.3 ± 0.01	0.3 ± 0.03	0.3 ± 0.01	0.30 ± 0.04	0.35 ± 0.01	0.37 ± 0.02	0.33 ± 0.01	0.36 ± 0.00	0.35 ± 0.00
TI	0.19 ± 0.01	0.2 ± 0.01	0.2 ± 0.01	0.2 ± 0.01	0.2 ± 0.03	0.2 ± 0.01	0.21 ± 0.02	0.22 ± 0.01	0.25 ± 0.03	0.21 ± 0.01	0.24 ± 0.01	0.23 ± 0.00
Omega 3	35.6 ± 1.9	36.7 ± 1.8	34.1 ± 0.8	33.6 ± 0.5	34.0 ± 3.0	35.6 ± 1.1	26.5 ± 3.5	32.5 ± 0.7	29.5 ± 2.5	32.4 ± 1.6	28.8 ± 1.9	30.7 ± 0.6
Omega 6	30.8 ± 1.1	30.0 ± 1.2	31.9 ± 0.6	29.9 ± 0.3	28.8 ± 0.6	29.1 ± 1.2	29.4 ± 3.2	29.5 ± 0.6	29.8 ± 0.6	31.6 ± 0.7	30.3 ± 0.5	30.2 ± 1.1
**Irrigation**	**Drought**											
**T and CO_2_**	**400 ppm**						**+4 °C, 700 ppm**					
**Inoculation**	**C**	**1**	**2**	**3**	**4**	**5**	**C**	**1**	**2**	**3**	**4**	**5**
SFA	24.2 ± 0.6	25.3 ± 1.2	24.8 ± 0.1	28.5 ± 2.4	24.9 ± 0.3	24.4 ± 0.5	26.0 ± 0.5	25.9 ± 0.7	26.8 ± 0.6	26.6 ± 0.7	26.3 ± 0.5	25.5 ± 0.5
MUFA	9.1 ± 0.6	8.4 ± 0.6	10.8 ± 1.0	10.7 ± 1.4	9.6 ± 0.4	10.7 ± 0.8	9.6 ± 0.7	12.4 ± 1.4	9.1 ± 1.3	9.3 ± 0.5	13.3 ± 0.5	11.5 ± 0.9
PUFA	66.7 ± 0.9	66.3 ± 1.6	64.4 ± 1.0	60.9 ± 3.6	65.5 ± 0.7	64.9 ± 1.1	64.4 ± 0.9	61.7 ± 2.1	51.0 ± 3.3	64.0 ± 1.0	60.4 ± 0.9	63.0 ± 1.3
PUFA/SFA	2.8 ± 0.1	2.7 ± 0.2	2.6 ± 0.1	2.2 ± 0.3	2.6 ± 0.1	2.7 ± 0.1	2.5 ± 0.1	2.4 ± 0.1	1.9 ± 0.2	2.4 ± 0.1	2.3 ± 0.1	2.5 ± 0.1
18:2/18:3	0.9 ± 0.03	0.8 ± 0.07	0.9 ± 0.06	0.9 ± 1.16	0.7 ± 0.04	0.8 ± 0.05	1.04 ± 0.10	1.15 ± 0.17	2.01 ± 0.25	1.08 ± 0.15	1.46 ± 0.12	1.03 ± 0.09
Cox	11.0 ± 0.2	11.2 ± 0.4	10.6 ± 0.3	9.8 ± 1.1	11.1 ± 0.1	10.9 ± 0.3	10.3 ± 0.1	9.8 ± 0.5	7.3 ± 0.6	10.2 ± 0.3	9.1 ± 0.3	10.1 ± 0.4
OS	4988.5 ± 89.1	5069.5 ± 190.4	4819.3 ± 136.3	4459.0 ± 503.6	5026.3 ± 59.1	4951.7 ± 131.6	4654.4 ± 63.4	4415.0 ± 248.2	3284.4 ± 295.5	4620.1 ± 166.6	4092.5 ± 123.9	4571.8 ± 174.8
h/H	3.3 ± 0.1	3.2 ± 0.1	3.3 ± 0.0	2.7 ± 0.3	3.2 ± 0.0	3.3 ± 0.1	3.0 ± 0.1	3.0 ± 0.1	2.4 ± 0.2	2.9 ± 0.1	3.0 ± 0.1	3.0 ± 0.1
PI	78.9 ± 1.5	81.5 ± 3.9	77.6 ± 2.4	70.4 ± 10.7	81.9 ± 1.3	81.0 ± 2.5	71.3 ± 2.2	69.2 ± 4.4	43.2 ± 5.9	70.6 ± 3.9	61.4 ± 2.5	71.9 ± 3.5
AI	0.3 ± 0.01	0.3 ± 0.02	0.3 ± 0.00	0.4 ± 0.05	0.3 ± 0.01	0.3 ± 0.01	0.35 ± 0.01	0.35 ± 0.01	0.46 ± 0.04	0.36 ± 0.01	0.36 ± 0.01	0.34 ± 0.01
TI	0.2 ± 0.01	0.2 ± 0.02	0.2 ± 0.01	0.3 ± 0.12	0.2 ± 0.00	0.2 ± 0.01	0.22 ± 0.01	0.24 ± 0.03	0.38 ± 0.04	0.23 ± 0.02	0.27 ± 0.02	0.22 ± 0.01
Omega 3	36.0 ± 0.9	37.7 ± 2.2	34.7 ± 1.7	31.1 ± 6.3	37.6 ± 0.8	36.7 ± 1.6	31.8 ± 1.2	29.6 ± 2.9	17.8 ± 2.7	31.5 ± 2.3	24.8 ± 1.5	31.4 ± 2.2
Omega 6	30.7 ± 0.4	28.6 ± 0.8	29.6 ± 0.7	29.8 ± 2.8	27.9 ± 0.8	28.2 ±0.9	32.6 ± 1.7	32.1 ± 1.0	33.2 ± 0.8	32.6 ± 1.5	35.6 ± 0.7	31.6 ± 1.0

**Table 2:** Generalized linear model (GLM) significance as *p*-values for FA and phenol and oxalate content of *S. ramosissima* shoots under conditions of T/CO_2_, irrigation, and PGPR inoculation (as categorical variables) and their interaction (*p* * < 0.05, *p* ** < 0.01, *p* *** < 0.001).

Treatment	FA 16:0	FA 18:0	FA 16:1t	FA 18:1	FA 18:2	FA 18:3	Phenols	Oxalate
T/CO_2_	0.0000 ***	0.0590	0.0000 ***	0.0000 ***	0.0000 ***	0.0000 ***	0.4109	0.0000 ***
Irrigation	0.1267	0.7633	0.0000 ***	0.1763	0.2043	0.0221 **	0.0000 ***	0.0000 ***
Inoculation	0.5454	0.3962	0.5359	0.0315 **	0.2587	0.0869	0.0966	0.0000 ***
T/CO_2_ × Irrigation	0.4179	0.0871	0.0005 ***	0.0262 **	0.0037 **	0.1464	0.0000 ***	0.3307
T/CO_2_ × Inoculation	0.4104	0.6916	0.0002 ***	0.0740	0.0011 **	0.0137 **	0.0075 **	0.0000 ***
Irrigation × Inoculation	0.1679	0.3482	0.1805	0.4864	0.5135	0.1859	0.0000 ***	0.0000 ***
T/CO_2_ × Irrigation × Inoculation	0.1088	0.7653	0.0174 *	0.3431	0.7529	0.0858	0.0000 ***	0.0000 ***

**Table 3:** Overview of the 36 treatments applied to *S. ramosissima* plants in our experiment.

CO_2_ and Temperature	Irrigation	Rhizobacteria Inoculation
400 ppm CO_2_ 25/14 °C	Optimal salinity (171 mmol L^−1^ NaCl)	None, 1, 2, 3, 4, and 5
	Salt stress (510 mmol L^−1^ NaCl)	None, 1, 2, 3, 4, and 5
	Drought	None, 1, 2, 3, 4, and 5
700 ppm CO_2_ 29/18 °C	Optimal salinity (171 mmol L^−1^ NaCl)	None, 1, 2, 3, 4, and 5
	Salt stress (510 mmol L^−1^ NaCl)	None, 1, 2, 3, 4, and 5
	Drought	None, 1, 2, 3, 4, and 5

**Table 4:** Isolation data and traits of the plant-growth-promoting rhizobacteria (PGPR) used in this study.

Consortia	Halophyte	Estuary and Year	Rhizobacteria Strain	Plant-Growth-Promoting Traits						Salt Tolerance (M NaCl)	Reference
				N Fixation	P Solubilization	Siderophores	IAA	Biofilm	ACC Deaminase		
1	*Spartina densiflora*	Tinto 2010	*Pseudomonas composti* SDT3		•	•				1.5	[83]
			*Aeromonas aquariorum* SDT13		•	•	•			0.5	
			*Bacillus thuringiensis* SDT14	•		•				1	
2	*Arthrocnemum macrostachyum*	Odiel 2015	*Vibrio kanaloae* RA1	•		•	•			0.6	[84]
			*Pseudoalteromonas prydzensis* RA15	•		•	•			1	
			*Staphylococcus warneri* RA18				•			1.5	
3	*Spartina maritima*	Tinto 2013	*Bacillus methylotrophicus* SMT38	•		•		•		2	[85]
			*Bacillus aryabhattai* SMT48	•	•	•	•			1.5	
			*Bacillus licheniformis* SMT51	•	•	•	•	•		2	
4	*Atriplex portulacoides*	Piedras 2017	*Vibrio spartinae* HPJ2	•	•	•	•	•	•	1	[37]
			*Marinobacter sediminum* HPJ15			•	•			1.7	
			*Vibrio parahaemolyticus* HPJ50	•	•		•	•		1	
5	*Salicornia ramosissima*	Tinto 2016	*Vibrio neocaledonicus* SRT1	•	•	•	•	•		1.7	[37]
			*Thalassospira australica* SRT8					•	•	1	
			*Pseudarthrobacter oxydans* SRT15	•	•		•			1	

## Data Availability

The data presented in this study are available on request from the corresponding author.
